# Efficient Production of Chimeric Hepatitis B Virus-Like Particles Bearing an Epitope of Hepatitis E Virus Capsid by Transient Expression in *Nicotiana benthamiana*

**DOI:** 10.3390/life11010064

**Published:** 2021-01-17

**Authors:** Gergana Zahmanova, Milena Mazalovska, Katerina Takova, Valentina Toneva, Ivan Minkov, Hadrien Peyret, George Lomonossoff

**Affiliations:** 1 Department of Plant Physiology and Molecular Biology, University of Plovdiv, 4000 Plovdiv, Bulgaria; mazalovska@yahoo.com (M.M.); katerina.takova@gmail.com (K.T.); toneva@plantgene.eu (V.T.); 2 Center of Plant Systems Biology and Biotechnology, 4000 Plovdiv, Bulgaria; minkov@cpsb.eu; 3 Institute of Molecular Biology and Biotechnologies, 4108 Markovo, Bulgaria; 4 Department of Biological Chemistry, John Innes Centre, Norwich Research Park, Colney NR4 7UH, UK; Hadrien.Peyret@jic.ac.uk

**Keywords:** hepatitis B capsid antigen, hepatitis E capsid protein, virus-like particles, chimeric HBcHEV VLPs, plant transient expression

## Abstract

The core antigen of hepatitis B virus (HBcAg) is capable of self-assembly into virus-like particles (VLPs) when expressed in a number of heterologous systems. Such VLPs are potential carriers of foreign antigenic sequences for vaccine design. In this study, we evaluated the production of chimeric HBcAg VLPs presenting a foreign epitope on their surface, the 551–607 amino acids (aa) immunological epitope of the ORF2 capsid protein of hepatitis E virus. A chimeric construct was made by the insertion of 56 aa into the immunodominant loop of the HBcAg. The sequences encoding the chimera were inserted into the pEAQ-*HT* vector and infiltrated into *Nicotiana benthamiana* leaves. The plant-expressed chimeric HBcHEV ORF2 551–607 protein was recognized by an anti-HBcAg mAb and anti-HEV IgG positive swine serum. Electron microscopy showed that plant-produced chimeric protein spontaneously assembled into “knobbly” ~34 nm diameter VLPs. This study shows that HBcAg is a promising carrier platform for the neutralizing epitopes of hepatitis E virus (HEV) and the chimeric HBcAg/HEV VLPs could be a candidate for a bivalent vaccine.

## 1. Introduction

Every year according to the World Health Organization, hepatitis E virus (HEV) causes approximately 20 million infections worldwide and 44,000 deaths related to hepatitis E. HEV infection is a self-limiting disease but can progress to chronic hepatitis. Pregnant women, as well as immunocompromised and immunosuppressed patients, are the high-risk groups with higher mortality rates and susceptibility to chronic HEV infection [1]. The mortality rate has been found to be around 20% in pregnant women, and up to 4% in the general population [2]. HEV is mainly transmitted by the fecal-oral route. Contaminated drinking water has been associated with several outbreaks in developing counties [3]. In developed counties, in the past decade, a number of autochthonous cases of HEV infection has been reported, and evidence for zoonotic transmission has been discovered [4]. The main HEV reservoirs are domestic pigs and wild boar [5,6]. Occupational exposure, as well as consumption of undercooked pork meat, are key factors for the spread of HEV in Europe [7,8]. A vaccine against the hepatitis E virus infection has been reported in China but is not yet available elsewhere [9].

HEV is a single-stranded, positive-sense RNA virus that belongs to the Hepeviridae family [10]. Seven mammalian genotypes have been identified (HEV 1–7) [11]. All seven genotypes of HEV are recognized as one serotype, making the development of a universal hepatitis E vaccine feasible. The HEV virion is small, non-enveloped with icosahedral symmetry and a size of 27–34 nm. The RNA genome contains three open reading frames (ORFs) [10]. ORF1 encodes the viral nonstructural polyprotein [12], ORF2 encodes the viral capsid protein [13], and ORF3 encodes a multifunctional protein [14]. All efforts aimed at the development of a hepatitis E vaccine have been focused on the ORF2 capsid protein [15]. The viral capsid protein consists of 660 amino acids and has the following three domains: S (shell, amino acids 112–319); M (middle, amino acids 320–455); and P (protruding, amino acids 456–606 [16]. The S domain assembles into a stable icosahedral shell, while the P domain protruding as a surface spike that is involved in host interactions and contains neutralization epitopes [17]. The immunodominant epitopes of ORF2 protein are located on the P domain and conserved among all HEV genotypes. The major anti-HEV antibody responses are against epitopes located at 459–606 aa of HEV ORF2 [18] and are mainly conformational [19]. Full-length and partially truncated versions of the capsid protein have been expressed in different systems (bacteria, yeast, insect cells, mammalian cells, and plants) to generate virus-like particles (VLPs) for vaccine design [20,21,22,23,24,25,26]. However, there can be issues of yield and product heterogeneity using this approach and alternatives are required.

VLPs are highly immunogenic structures and can induce protective immunity. The hepatitis B capsid protein (HBcAg) has been shown to be capable of self-assembly into VLPs when expressed in a number of eukaryotic heterologous systems including mammalian cells [27], plants [28,29,30], yeast [31], insect cells [32], and Xenopus oocytes [33]. VLPs composed of HBcAg are safe and potent vaccine delivery systems [34,35]. HBcAg consists of 183 amino acids with an N-terminal assembly domain (1–149 aa) and a C-terminal arginine-rich domain (34 aa) required for the packaging of nucleic acid [36]. HBcAg is a homodimer that can self-assemble into icosahedral capsid with T = 4 (120 dimers) and T = 3 (90 dimers) symmetry and a size of 34 nm and 30 nm [37]. The HBcAg VLPs are very strong immunogens and give both a T-cell dependent and a T-cell independent antibody response [38]. Insertion of foreign sequences into the immunodominant c/e1 B cell epitope, a surface-exposed loop on the HBV capsid protein, results in particles that have the antigenic characteristics of HBcAg and the immunogenicity of the inserted foreign epitope which is considerably enhanced. These make HBcAg one of the most promising delivery vehicles for delivering specific epitopes, such as those from HEV, to the immune system for potent vaccine production [39,40,41]. 

Amino acids 551–607 from HEV ORF2 are immunogenic epitopes from the main immunogenic region of the ORF2. To improve the immunogenicity of this sequence, HBcAg VLPs have been used as particulate carriers. Here, we describe the efficient expression in *Nicotiana benthamiana* plants of chimeric HBcAg VLPs carrying this epitope and evaluate the particles as candidate HEV vaccines. Transient expression with the pEAQ-*HT* vector of a HBcHEV ORF2 551–607 construct led to the production of chimeric VLPs. We examined the ultrastructure of the particles by electron microscopy and showed that they consisted of particles 28–38 nm in diameter, typical of HBcAg VLPs, with a “ragged” surface, which was most likely due to the location of the HEV ORF2 56 aa on the top of the HBc spikes. Furthermore, the chimeric protein was recognized by an anti-HEV IgG positive swine serum and retained HBcAg reactivity. From this study, we believe that HBcHEV ORF2 551–607 protein could be used for future immunological studies. 

## 2. Materials and Methods

### 2.1. Gene Cloning and Plasmids Construction

The 56 amino acid residues of HEV ORF 2 551–607 consensus sequences were based on HEV genotype 3 nucleoprotein HEV ORF2 1–660 (GenBank accession number DQ079627.1). Amplification of DNA fragment for the production of the HEV ORF2 501–607, was carried out by PCR. The target HEV ORF2 551–607 sequences were amplified using the synthetic master gene HEV ORF2 1–660 [25]. The PCR fragment was flanked by *Sal*I and *Ase*I restriction sites (New England Biolabs, Ipswich, MA, USA), which were used for sub-cloning of the PCR fragment. The HEV ORF2 551–607 peptide was cloned into the MIR of the monomeric HBcAg construct in the previously described pEAQ-mEL [42]. The resulted vector was named pEAQ-HT:HBcHEV ORF2 551–607. Following the heat-shock transformation of competent *Escherichia coli* XL1-Blue, putative clones harboring the expression vector (pEAQ-*HT*:HBcHEV ORF2 551–607) were verified by sequencing (Eurofins, Hamburg, Germany). 

### 2.2. Agrobacterium tumefaciens Transformation and Nicotiana benthamiana Agroinfiltration 

The recombinant vector pEAQ-*HT*:HBcHEV ORF2 551–607 was transformed into the electrocompetent *A. tumefaciens* strain LBA4404. Then, transformed colonies were selected from agar plates containing 50 μg/mL kanamycin and 50 μg/mL rifampicin. Agrobacterial suspensions were cultured at 28 °C and shook (200 rpm) for 48 h. The agrobacterial cells were pelleted by centrifugation (4000× *g* centrifugation for 10 min.). Following resuspension in 10 mM MES 2-(N-morpholino) ethansulfonic acid, pH 5.6, 10 mM MgCl_2_ and 100 µM acetosyringone to an OD600 of 0.4 and incubation of 3 h at room temperature, bacterial suspensions were syringe-infiltrated into the leaves of 5–6-week-old *N. benthamiana* plants. Leaf tissue was harvested 7 days post infiltration (dpi). Control agrobacterial suspensions containing the empty pEAQ-*HT* vector [43] without a gene insert and with pEAQ-*HT*:HBcAg were included to compare with pEAQ-*HT*:HBcHEV ORF2 551–607 for observation of protein expression and VLPs formation.

### 2.3. Protein Extractions, SDS-PAGE, and Western Blot Analyses

Small-scale protein extraction was conducted to test for HBcHEV ORF2 551–610, and mHBAg expression and accumulation. Approximately 90 mg of the infiltrated leaf was harvested at 7 dpi and homogenized with a ceramic bead for 30 s at speed 4 (MP Biomedicals, Irvine, CA, USA) in 3× volume of extraction buffer (100 mM sodium phosphate at pH 6.8, 150 mM NaCl, 0.1% Triton-X and protease inhibitors (Roche, Basel, Switzerland)). Then, samples were centrifuged at 13,000× *g* for 10 min and the supernatant was kept as soluble protein fraction. Nevertheless, the insoluble protein (IP) fraction could be extracted from the pellet through boiling with protein denaturing buffer (NuPAGE LDS buffer (Life Technologies, Carlsbad, CA, USA) mixed 3:1 with 2-mercaptoethanol) and centrifuged at 13,000× *g* for 10 min. Proteins were analyzed by sodium dodecyl sulfate-polyacrylamide gel electrophoresis (SDS-PAGE) on 12.5% (*w*/*v*) polyacrylamide-SDS or NuPAGE Bis-Tris gels (Life Technologies, Carlsbad, CA, USA) and the gels stained with InstantBlue (Merck, St. Louis, MO, USA). For Western blot analysis, the electrophoresed proteins were electroblotted onto a nitrocellulose membrane (GE Healthcare, Chicago, IL, USA) via wet transfer at 100 V for 1 h. Blotted membrane was blocked with 5% (*w*/*v*) milk powder in phosphate-buffered saline (PBS) containing 0.05% (*v*/*v*) Tween 20 (PBST) for at least 1 h. The membrane was subsequently incubated with either mouse anti-HBcAg monoclonal antibody (10E11, Abcam, Cambridge, UK) (1:4000 dilution) or HEV immunoglobulin G (IgG) positive swine serum (dilution 1:200) for 1 h. The membrane was washed three times with PBST at 5 min intervals. Then, the membrane was incubated with a goat anti-mouse IgG horseradish peroxidase (HRP)-conjugated secondary antibody (Thermo Fisher Scientific, Waltham, MA, USA) diluted 1:30,000 for detection of 10E11, and secondary anti-pig IgG antibody-HRP (Thermo Fisher Scientific, Waltham, MA, USA) diluted 1:8000 for detection of swine serum, respectively. Washing steps were repeated three times before the blot was soaked in SuperSignal™ West Dura Extended Duration Substrate (Thermo Fisher Scientific, Waltham, MA, USA). The emitted luminescence was detected with the ImageQuant LAS 500 system (GE Healthcare, Chicago, IL, USA) and on X-ray film. 

### 2.4. Indirect Enzyme-Linked Immunosorbent Assay (ELISA)

Microtiter plates (Greiner 96-well flat bottom) were coated with 100 µL/well of serial dilutions of purified protein in duplicate wells (100 ng/well to 12.5 ng/well) in PBS (pH 7.4) and incubated overnight at 4 °C. After three washes with PBST, plates were incubated with 200 µL/well of blocking solution (PBST 5% (*w*/*v*) dry milk) and incubated for 1 h at room temperature. Then, 100 µL anti-HEV IgG positive and negative swine serum diluted 1:100 in blocking buffer was added and incubate for 1 h at 37 °C. Plates were washed again with PBST before the addition of 100 µL/well of an HRP-conjugated anti-swine secondary antibody 1:10,000 (Thermo Fisher Scientific, Waltham, MA, USA). After incubation with the secondary antibody, plate wells were washed three times before adding 50 µL/well of the substrate solution of OPD Substrate Tablets (Thermo Fisher Scientific, Waltham, MA, USA). Plates were incubated, in the dark, at room temperature from 10 to 20 min, and then the reaction was stopped by the addition of 50 µL/well of 1M H_2_SO_4_, and the plates were read at 492 nm in a plate reader Epoch Microplate Spectrophotometer (BioTek Instruments Inc., Winooski, VT, USA). Previously characterized negative swine sera by using commercial kits from PrioCheck HEV Ab (Thermo Fisher Scientific, Waltham, MA, USA) were used as controls.

### 2.5. Purification of Virus-Like Particles (VLPs)

To purify assembled particles derived from constructs pEAQ-*HT*:HBcHEV ORF2 551–607 and pEAQ-*HT*:HBcAg, leaf tissue was harvested at 7 dpi and homogenized in three volumes of the same extraction buffer. Crude extracts were filtered through a layer of Miracloth before subjecting to centrifugation (20,000× *g*) at 4 °C for 20 min using an SS34 rotor (Thermo Fisher Scientific, Waltham, MA, USA). The clarified supernatant was filtered over 0.45 µm syringe filters. The VLP samples were subjected to a two-stage purification process, as described by Peyret 2015 [44]. Firstly, clarified extracts were overlain above two different concentrations of sucrose solution, 6 mL of 25% (*w*/*v*) and 2 mL of 70% (*w*/*v*). The sucrose cushions were prepared in UltraClear ultracentrifuge tubes (Beckman Coulter, Brea, CA, USA) and centrifuged in a Surespin 630/36 rotor (Thermo Fisher Scientific, Waltham, MA, USA) at 167,000× *g*, 4 °C, for 2.5 h. The gradient was fractionated by piercing the bottom of the tube with a needle and recovering the bottom and interface fractions. Then, these fractions were dialyzed thoroughly against 200 mM ammonium bicarbonate buffer (pH 8.0) overnight. The samples were concentrated using a SpeedVac vacuum concentrator (Thermo Fisher Scientific, Waltham, MA, USA), and loaded onto a Nycodenz step gradient (SERVA, Merelbeke, Belgium) extending from 60 to 20% (*w*/*v*), with 2 mL of each concentration in UltraClear ultracentrifuge tubes (Beckman Coulter, Brea, CA, USA). High-speed centrifugation was operated at 274,000× *g* using the TH-641 rotor (Thermo Fisher Scientific, Waltham, MA, USA) for 20 h, at 4 °C. The mHBcAg and mHBcAg-HEV ORF2 551–607 proteins were dialyzed against PBS using a Float-A-Lyzer (MilliporeSigma, St. Louis, MO, USA).

### 2.6. Agarose Gel Electrophoresis

To determine if particles incorporated RNA, equal protein amounts of purified mHBcAg VLPs and chimeric HBcHEV ORF2 551–607 VLPs were loaded on 1% of agarose gel. The RNA content of the different protein fractions was analyzed on ethidium bromide-stained 1% agarose gels followed by Coomassie blue staining.

### 2.7. Transmission Electron Microscopy (TEM) of Purified VLPs

Approximately 10 µL of the VLPs sample was adsorbed on copper-palladium grids, washed with sterile distilled water, and negatively stained with 2% (*w*/*v*) uranyl acetate. As an experimental control, the monomer HB core particles without a gene insert were also examined. The grids were examined using an FEI Tecnai 20 transmission electron microscope (FEI, Lausanne, Switzerland).

## 3. Results

### 3.1. Gene Design

**Figure 1:**
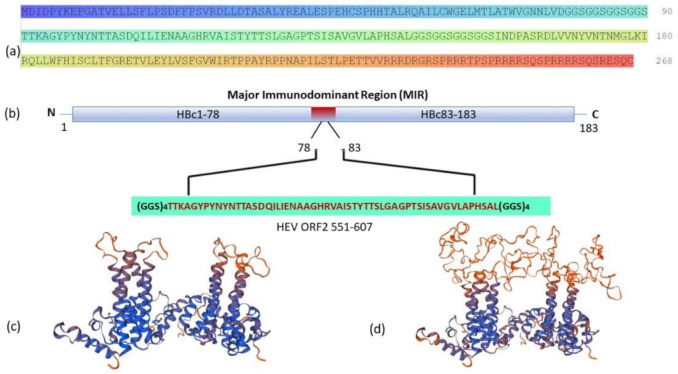
Structure of the chimeric HBcHEV ORF2 551–607 construct. (**a**) Amino acid sequences of chimeric HBcHEV ORF2 551–607 construct; (**b**) Schematic representation of HBcAg with HEV ORF2 551–607 immunogenic epitope inserted between 78 aa and 83 aa, with a (GGS)4 linker at both ends; (**c**) Predicted stucture of HBcAg; (**d**) Predicted structure of chimeric HBcAg presenting the HEV ORF2 551–607 epitope in the c/e1 immunodominant loop (MIR), as was done by SWISS_MODEL [46].

According to Peyret (2015) [45], we constructed a fusion protein by inserting 56 aa of ORF2 551–6107 antigenic peptide from hepatitis E virus between amino acids 78 to 83 on the c/e1 immunogenic loop of HBcAg (Figure 1, panel b). In this construct, the ORF2 551–6107 antigenic peptide is linked to HBcAg with three amino acid linkers ((GGS)4) on either side. The predicted three-dimensional (3D) structure of HBcHEV ORF2 551–607 chimeric protein shows that the ORF2 56 aa is on the top of the HBc spikes (Figure 1, panel d).

### 3.2. Gene Cloning

**Figure 2:**
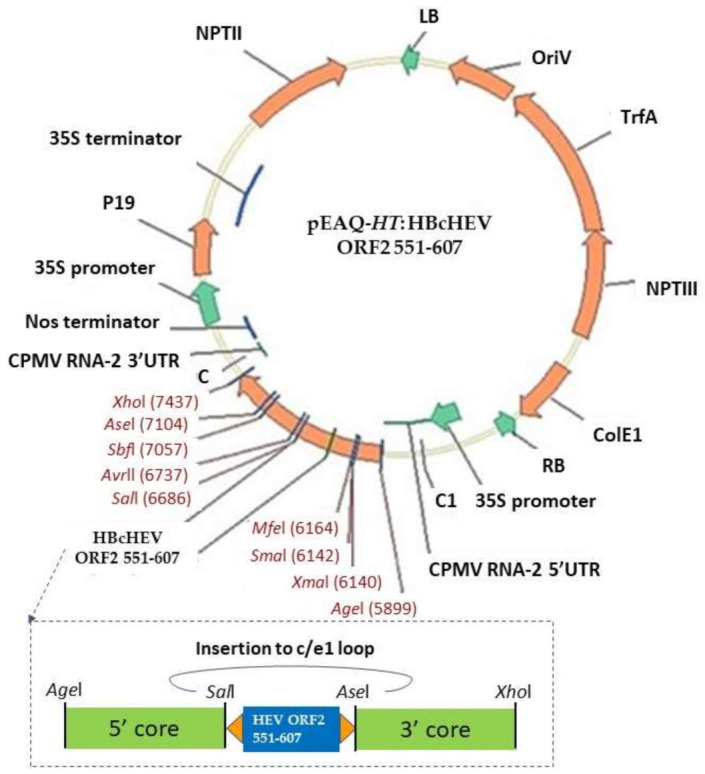
Illustrates the expression cassette and the corresponding recombinant vector used in this study.

The nucleotide sequences of the whole HEV genotype 3 nucleoprotein (HEV ORF2 1–660) [25] codon-optimized for *N. benthamiana* were used as templates for PCR amplification of HEV ORF2 551–607 to construct the pEAQ-HT:HBcHEV ORF2 551–607 (Figure 2). Primers were designed to incorporate SalI and AseI restriction sites and long flexible linkers ((GGS)4) at 5’ and 3’ ends of the HEV ORF2 551–607 region. Then, the amplified HEV ORF2 551–607 fragment was cloned into the MIR region of the monomeric HBcAg construct in the previously described pEAQ-mEL [42], referred to in this study as a pEAQ-HT:HBcAg. 

The constructs pEAQ-HT:HBcAg and pEAQ-HT:HBcHEV ORF2 551–607 were transformed into *A. tumefaciens* and used for expression of the recombinant proteins in *N. benthamiana*.

### 3.3. Expression of Recombinant pEAQ-HT:HBcAg and pEAQ-HT:HBcHEV ORF2 551–607 Constructs in N. benthamiana

**Figure 3:**
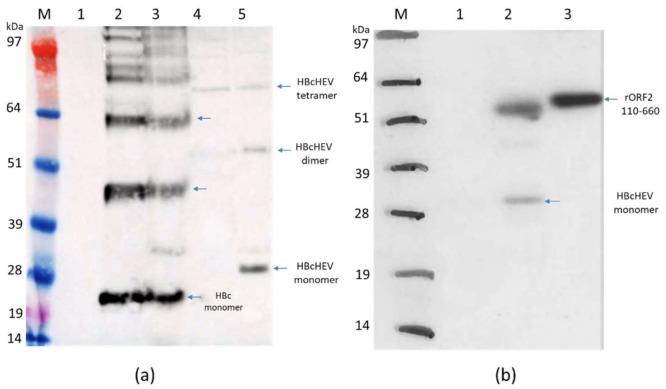
Western blot analysis of HBcAg and HBcHEV ORF2 551–607. (**a**) Western blot with mouse anti-HBcAg monoclonal antibody (10E11, Abcam, UK). M, marker; 1, pEAQ-HT empty vector; 2, HBcAg supernatant (SN) after extraction in 3x volume extraction buffer and 13k rpm; 3, HBcAg pellet; 4, HBcHEV ORF2 551–607 SN; 5, HBcHEV ORF2 551–607 pellet; (**b**) Western blot with polyclonal anti-HEV IgG swine serum. M, marker; 1, pEAQ-HT empty vector; 2, HBcHEV ORF2 551–607 crude extract; 3, positive control rHEV 110–660. Arrows indicate monomer, dimer, and tetramer. SeeBlue Plus2 Pre-stained Protein Standard was used as a molecular marker.

*N. benthamiana* leaves agroinfiltrated with the recombinant vector pEAQ-HT:HBcAg and pEAQ-HT:HBcHEV ORF2 551–607 were harvested on the 7th day post infiltration (dpi) and analyzed by SDS-PAGE and Western blot to check protein expression. Western blot analysis with an anti-HBc mAb (10E11) (Figure 3, panel a.) confirmed that the HBcAg and HBcHEV ORF2 551–607 proteins had been expressed well in *N. benthamiana* plants. Expression of the pEAQ-HT:HBcAg in plants gave a monomeric form ~21 kDa protein, and a ladder of further immunoreactive proteins, consistent with being dimers, tetramers, and higher forms. The HBcHEV ORF2 551–607 protein was observed as a monomer with size ~29 kDa and a dimer with size ~56 kDa (Figure 3, panel a, lane 5). From the Western blot data (Figure 3, panel a), it appears that most of the HBcAg proteins are soluble. Furthermore, the yield of soluble HBcHEV ORF2 551–607 (Figure 3, panel a, lane 4) was low as compared with the insoluble fraction (Figure 3, panel a, lane 5). Immunoblot analyses with positive anti-HEV IgG swine sera showed that the antigenicity of the HEV ORF2 551–607 peptide was maintained and chimeric protein of HBcHEV ORF2 551–607 was recognized by polyclonal anti-HEV IgG Ab in swine sera (Figure 3, panel b). The polyclonal anti-HEV antibodies revealed the monomer form (~29 kDa) and the dimer form (~56 kDa) of the recombinat HBcHEV ORF2 551–607. As a positive control (Figure 3, panel b, lane 3), we used the rHEV 110–660 (~58 kDa) produced in plants from our previous study [25].

### 3.4. Purification of HBcAg VLPs and Chimeric mHBc VLPs Presenting the HEV ORF2 551–607 Epitope

**Figure 4:**
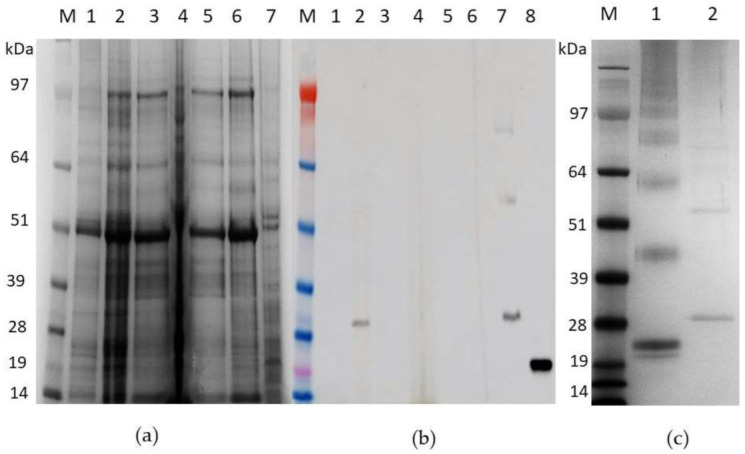
Gradient purification and detection of HBcHEV ORF2 551–607 virus-like particles (VLPs). (**a**) SDS-PAGE analysis of fractionated samples from the sucrose cushions, the gel was stained with InstantBlue; (**b**) Western blot profiles showing that the VLPs were successfully detected in the 70% sucrose and interface fractions using HBcAg monoclonal antibody 10E11. M, marker; 1, pEAQ-HT; 2, crude extract; 3, supernatant; 4, pellet; 5, ultracentrifugation supernatant; 6, 25% sucrose gradient fraction; 7, interface and 70% sucrose gradient fraction; 8, positive control recombinant HBcAg (Jena BioSsience, Germany); (**c**) SDS-PAGE of fractioned VLPs from the Nycodenz gradients. M, marker; 1, fractioned HBcAg VLPs; 2, fractined HBcHEV ORF2 551–607 VLPs. SeeBlue Plus2 Pre-stained Protein Standard was used as a molecular marker.

For large-scale purification, five plants per construct were infiltrated with either pEAQ-HT:HBcAg or pEAQ-HT:HBcHEV ORF2 551–607 and harvested at 7 dpi. The first purification step via sucrose cushion yielded 70% sucrose and interface fractions, which were collected for maximal recovery of VLPs present in the sample. The profiles of fractionated HBcHEV ORF2 551–607 samples are shown in Figure 4. The presence of HBcHEV ORF2 551–607 VLPs in both 70% sucrose and interface was confirmed via Western blotting (Figure 4, panel b, lane 7).

These VLP-containing fractions were pooled and subjected to a Nycodenz gradient for further purification. The Nycodenz gradient of HBcAg gave rise to two obvious bands (Figure S1, panel a) and a single grayish band of HBcHEV ORF2 551–607 (Figure S2, panel a). The bands were collected with a needle and syringe and subjected to SDS-PAGE analysis, followed by staining with InstantBlue (Figure 4, panel c). 

### 3.5. Transmission Electron Microscopy

**Figure 5:**
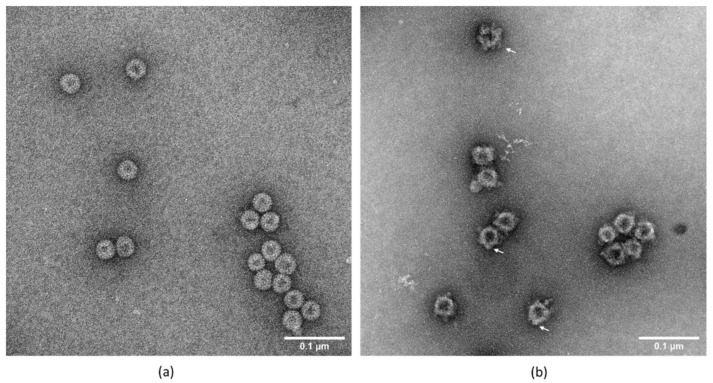
Electron micrograph showing the purified HBcAg and HBcHEV ORF2 551–607 VLPs that were negatively stained with 2% (*v*/*v*) uranyl acetate. (**a**) HBcAg VLPs; (**b**) HBcHEV ORF2 551–607. Scale bar = 0.1 µm; The chimeric HBcAg VLPs with HEV ORF2 551–607 epitope had successfully assembled into viral particles with “knobbly” structure. Arrows indicate “knobbly” VLPs.

Transmission electron microscopy revealed that plant produced native HBcAg protein and chimeric HBcHEV ORF2 551–607 protein had successfully assembled into VLPs (Figure 5). Native HBcAg assembled into spherical particles with an average size of 29.6 nm (+/− standard error 1.5) nm in diameter. The observed chimeric VLPs had an irregular spherical morphology probably due to the insertion of a 56 aa from ORF2 protruding peptide into the c/e1 loop. The diameters of chimeric VLPs ranged from 28 to 38 nm, the average size of chimeric VLPs was ∼34.1 nm in diameter with a standard error (+/− 0.7). As compared with the uniformly shaped HBcAg VLPs (Figure 5, panel a), the chimeric HBcAg VLPs seemed to exhibit an uneven surface layer, known as “knobbly” VLPs due to the protruding of ORF2 551–607 epitope from HBcAg spikes (Figure 5, panel b).

### 3.6. Indirect ELISA for HBcHEV ORF2 551–607 VLPs Detection

**Figure 6:**
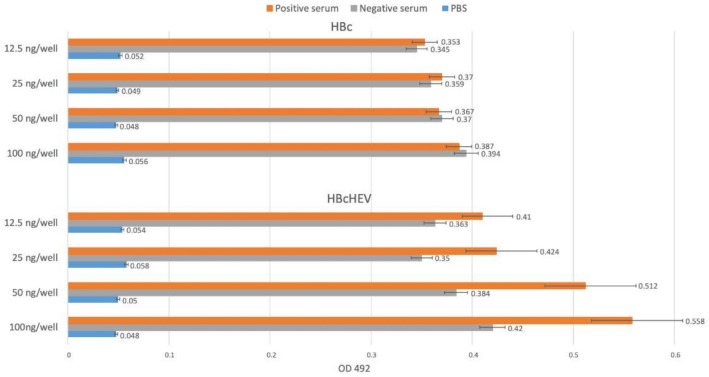
Indirect ELISA based on the HBcHEV ORF2 551–607 VLPs as a coating protein and the HBc VLPs as a control of specific binging of the positive anti-HEV Ig G swine serum. Previously characterized negative swine sera were used as controls. On the x axis are the concentration of the recombinant protein in the well, and on the y axis are the optical density 492 nm value. Data are shown as absorbance values of the used serum samples, the bars refer to standard error. The presented absorbance values are average values with standard errors calculated from four different negative serums and four different positive serums, repeated in two independent experiments.

Recognition of chimeric HBcHEV ORF2 551–607 VLPs from polyclonal anti-HEV Ig G swine serum was demonstrated by an indirect ELISA using wells coated with the HBc HEV chimeric particles. Anti-HEV IgG positive swine serum was used for detection of HBcHEV ORF2 551–607 VLPs. The ELISA plate was coated with different concentrations of HBcHEV ORF2 551–607 VLPs (100 ng/well, 50 ng/well, 25 ng/well, and 12.5 ng/well) or HBcAg at the same concentrations as controls for specific interactions. An anti-HEV IgG negative swine serum was used as a control for background signal. The anti-HEV Ig G positive swine serum specifically binds the HBcHEV ORF2 551–607 VLPs, when the concentration of the VLPs is 100 ng/well (Figure 6). The absorbance value of the positive serum is 0.558 (95% CI 0.517 to 0.599) as compared with the negative serum with OD 0.42 (95% CI 0.398 to 0.442), the p-value is 0.050004. Thus, the result is significant at *p* = 0.05.

### 3.7. Nucleic Acid Content Analysis of VLPs

**Figure 7:**
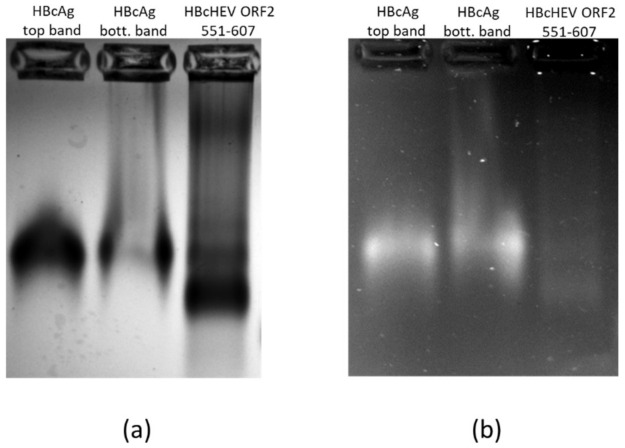
Agarose (1% *w*/*v*) gel electrophoresis of top and bottom band of gradient purified HBcAg VLPs and chimeric HBcHEV ORF2 551–607 VLPs. (**a**) Coomassie blue staining; (**b**) ethidium bromide (0.4 μg/mL) staining.

A purified chimeric HBcHEV ORF2 551–607 VLPs and HBcAg VLPs fractions were loaded on an agarose gel and after electrophoresis, the gel was stained with ethidium bromide (EtBr) followed by staining with Coomassie blue for identification of nucleic acids incorporation into VLPs. The VLP fractions migrated as a band that stained positive in either EtBr (Figure 7, panel b) or Coomassie blue staining (Figure 7, panel a). As expected, both wild-type and chimeric particles contained RNA/DNA. The RNA/DNA binding site in the HBcAg and HBcHEV ORF2 551–607 was not modified and internal RNA/DNA was confirmed by EtBr staining (Figure 7, panel b).

## 4. Discussion

The development of a recombinant vaccine against HEV, in which the ORF2 capsid protein or neutralizing epitopes of ORF2 are presented as multimeric structures, is highly desirable. Here, we present a strategy for vaccine design and characterization of the HEV ORF2 551–607 epitopes that may be useful for further HEV vaccine design. The HEV ORF2 551–607 peptide DNA sequences were synthesized by PCR and cloned into HBcAg coding sequenced between amino acid 78 to 83. The HBcAg gene and HEV ORF2 551–607 sequences expressed in this study were codon-optimized based on *N. benthamiana* preference to boost translation by using codons preferred by the host expression system. In addition, (GGS)4 linkers were inserted between HBcAg and HEV ORF2 551–607, to stabilize the conformation of the chimeric VLPs and compose the multidomain protein. 

The inserted HEV ORF2 551–607 sequences into HBc immunodominant loop (between 73 and 83 aa) is expected to be displayed on the HBcAg VLP surface. Protein modeling analysis of the chimeric VLPs (Figure 1, panel d) showed that HEV ORF2 551–607 epitope was exposed on the surface of the HBcAg. HEV positive swine serum recognized the chimeric proteins (Figure 3, lane b and Figure 6) that showed recognition of HEV ORF2 551–607 peptide from polyclonal anti-HEV IgG antibodies. The resulting chimeric HBcAg VLPs were expected to display a high density of HEV ORF2 551–607 epitopes on 90 or 120 copies of HBcAg protein dimers per particle. 

In many cases, the immunogenicity of small synthetic peptides can be increased after being attached to a carrier molecule such as VLPs. In a previous study, HBcAg carrying linear epitope located at 423–437 aa could self-assemble into VLPs and these particles could induce anti-ORF2 Abs, but the synthetic 423–437 aa peptide could not [18]. This study supported our idea that the HBcHEV ORF2 551–607 could be a better antigen and could enhance the immunogenicity of ORF2 551–607 aa and induce a strong antibody response. Moreover, a previous study [18] utilized a non-immunodominant linear neutralizing epitope against the hepatitis E virus.

Because HBcAg is an immunodominant immunogen [47], and insertion into c/1e epitope results in “inheritance“ of the immunodominance of the insertion, we inserted the ORF2 551–607 aa peptide into the c/1e loop. Glycine/serine-rich linkers were included between the HEV ORF2 551–607 aa and core protein to avoid a spurious interaction. This assay resulted in a chimeric VLP antigen, which could be used to prove the ORF2 551–607 aa activity. These chimeric VLPs could mimic the neutralizing epitope of HEV and provide a novel tool for subunit vaccine design. Expression of chimeric HBcHEV ORF2 551–607 protein into *N. benthamiana* does not lead to necrosis or apoptosis (data not shown) which allowed us to harvest the plants at the peak of recombinant protein accumulation. In this case, the optimal harvest time for the chimeric HBcHEV ORF2 551–607 VLPs was determined to be around 7 dpi. During this study, a constant OD 600 of 0.4 for the agrobacteria infiltration suspension was used, as reported previously [48]. HBcAg construct and chimeric construct produced monomer, dimer, and tetramer forms of the recombinant proteins during the expression in *N. benthamiana* (Figure 3 and Figure 4, panel c), we also observed multimers above that, classic with plant-produced HBcAg constructs [49]. A two-step protocol was used for VLP purification [44]. The first step was a discontinuous 20% and 70% sucrose cushion to enrich the isolation of VLPs, and the second step was an additional isopycnic (Nycodenz) gradient which was applied for preparation of high purity particles. The two-step protocol gave us a clear picture of HBcAg VLPs in two visible bands on the Nycodenz gradient (Figure S1, panel a) and chimeric HBcHEV ORF2 551–607 VLPs in one visible band (Figure S2, panel a). Their lower density and the apparent ability of stain to penetrate them suggests that VLPs from the upper band in Figure S1 may contain less RNA than those from the lower band. The chimeric particle diameter ranged from 28 to 38 nm, and the surface appeared to be rather “knobbly” (Figure 5, panel b). The “knobbly” surface morphology is expected for HBcAg core particles displaying a heterologous sequence on their surface [34,42]. This indicates that HEV ORF2 551–607 aa were presented on the surface of HBcAg spikes. The chimeric particles appeared to be larger than the HBcAg VLPs with diameters around 29 nm (Figure 5). The fact that the anti-HEV IgG positive sera recognizes the HBcHEV ORF2 551–607 particles indicate that the 56 aa from HEV is inserted on the surface of the particles, and therefore supports the idea that HBcAg can be used as a platform for HEV vaccine production.

The VLPs of HBcAg produced in *N. benthamiana* plants have been reported to encapsidate random cellular nucleic acid [42]. The presence of Arg-rich C-terminal domain into the chimeric HBcHEV ORF2 551–607 protein retains its ability for nucleic acid encapsulation into VLPs (Figure 7). However, the encapsidated nucleic acids have the advantage of producing a better antibody response against the insert as a result of the integral toll-like receptors (TLR) signaling [27,50].

Transient expression of recombinant proteins in *N. benthamiana* plants has emerged as an alternative to other expression systems such as yeast, bacterial, and mammalian cells. Plant expression systems have many advantages in terms of safety as plant pathogens do not infect humans and also owing to their scalability and their ability to perform a eukaryotic post-translational modification [51]. Some limitations such as post-synthesis instability, proteolytic degradation of the recombinant proteins, and product heterogenicity must be overcome in plant expression systems [52]. In our previous study, we successfully expressed modified versions of the ORF 2 capsid protein in *N. benthamiana* plants [25]. Plants expressed the HEV ORF2 proteins up to 10% of total soluble protein (TSP), but the efficiency of VLPs isolation through gradient centrifugation was very low. In the present study, the expression level of the recombinant HBcHEV ORF2 551–607 protein is lower, but the efficiency of particle formation and purification is higher, and the recovery yields of VLPs is approximately 10 mg/kg green leaf biomass. The recovered purified yield of HBcAg was found to be five to ten times higher than the yield of HBcHEV ORF2 551–607. In order to deliver the ORF2 VLPs in sufficient amounts for them to be evaluated cost-effectively in animal model trials, robust manufacturing strategies need to be put into place. The HBcAg provides a flexible strategy for the design of chimeric particles, and the use of the pEAQ vector based on genetic elements of CPMV genome expression system provides efficient expression and particle formation [53].

Currently, three HEV vaccine candidates have been under evaluation in clinical trials. Two of them have been produced in *E. coli* as VLPs, including p179 and p239 [15], the third one was produced in insect cell as a 56 kDa recombinant protein [54]. Developing a vaccine for HEV in plants, we are competing with *E. coli* and the baculovirus-insect cell system. In our previous studies, we showed that the HEV ORF2 110–610 recombinant protein formed unstable nanosized virus-like particles with non-uniform sizes ranging from 10 to 100 nm [22,25]. The immunization of mice with plant-derived HEV 110–610 unstable VLPs induced high levels of HEV-specific serum antibodies [22] demonstrating the immunogenicity of plant-derived recombinant proteins. However, it is necessary to look for methods for obtaining stable HEV VLPs in plants, and the HBc HEV VLPs address this need. 

## 5. Conclusions

We report the expression and the self-assembly of chimeric HBcHEV ORF2 551–607 VLPs in the plants, representing the first report of the successful transient expression of chimeric HBV/HEV VLPs in this expression system. Hence, plants may be a novel source for the cost-effective production of multivalent vaccines. Currently, we do not know if the HEV ORF2 551–607 epitope is a neutralizing epitope. Future studies of the immunogenicity of chimeric HBV/HEV ORF2 VLPs in animal models and their ability to protect against HEV infection are ongoing.

## Data Availability

No new data were created or analyzed in this study.

## Supplementary Materials

The following are available online at https://www.mdpi.com/2075-1729/11/1/64/s1, Figure S1: Nycodenz gradient purification and transmission electron microscopy (TEM) for the detection of HBcAg VLPs. (**a**) Visualization of two bands in Nycodenz gradient by downward illumination of the tube; (**b**) Negative stain TEM of the top band from Nycodenz gradient; (**c**) Negative stain TEM of bottom band from Nycodenz gradient. Scale bar = 100 nm. Arrows indicate smaller particles, Figure S2: Nycodenz gradient purification and transmission electron microscopy (TEM) detection of HBcHEV ORF2 551–607 VLPs. (**a**) Visible single band of VLPs observed from Nycodenz gradient; (**b**) TEM imaging of the collected single band. Particles were visualized by negative staining with uranyl acetate. Scale bar = 100 nm.
