# Supplementary material for: Efficient Production of Chimeric Hepatitis B Virus-Like Particles Bearing an Epitope of Hepatitis E Virus Capsid by Transient Expression in Nicotiana benthamiana
Source: Life (Basel). 2021 Jan 17;11(1):64. doi: 10.3390/life11010064 (PMC7830250; doi:10.3390/life11010064)
Supplement: Supplementary file 1 [file life-11-00064-s001.pdf]

life

MDPI

Article

Efficient Production of Chimeric Hepatitis B Virus-Like Particles Bearing an Epitope of Hepatitis E Virus Capsid by Transient Expression in *Nicotiana Benthamiana*

Gergana Zahmanova [1,2,*], Milena Mazalovska [1], Katerina Takova [1], Valentina Toneva [1,3], Ivan Minkov [2,3], Hadrien Peyret [4] and George Lomonossoff [4,*]

[1] Department of Plant Physiology and Molecular Biology, University of Plovdiv, Plovdiv, 4000, Bulgaria; gerganaz@uni-plovdiv.bg
[2] Center of Plant Systems Biology and Biotechnology, Plovdiv, 4000, Bulgaria; minkov@cpsb.eu
[3] Institute of Molecular Biology and Biotechnologies, Plovdiv, 4000, Bulgaria; toneva@plantgene.eu
[4] Department of Biological Chemistry, John Innes Centre, Norwich Research Park, Colney NR4 7UH, UK; george.lomonossoff@jic.ac.uk
* Correspondence: (G.Z.); george.lomonossoff@jic.ac.uk (G.L.); Tel.: +359 32 261529 (G.Z.); Tel.: + 44 1603 450351 (G.L.)

Supplementary Materials:

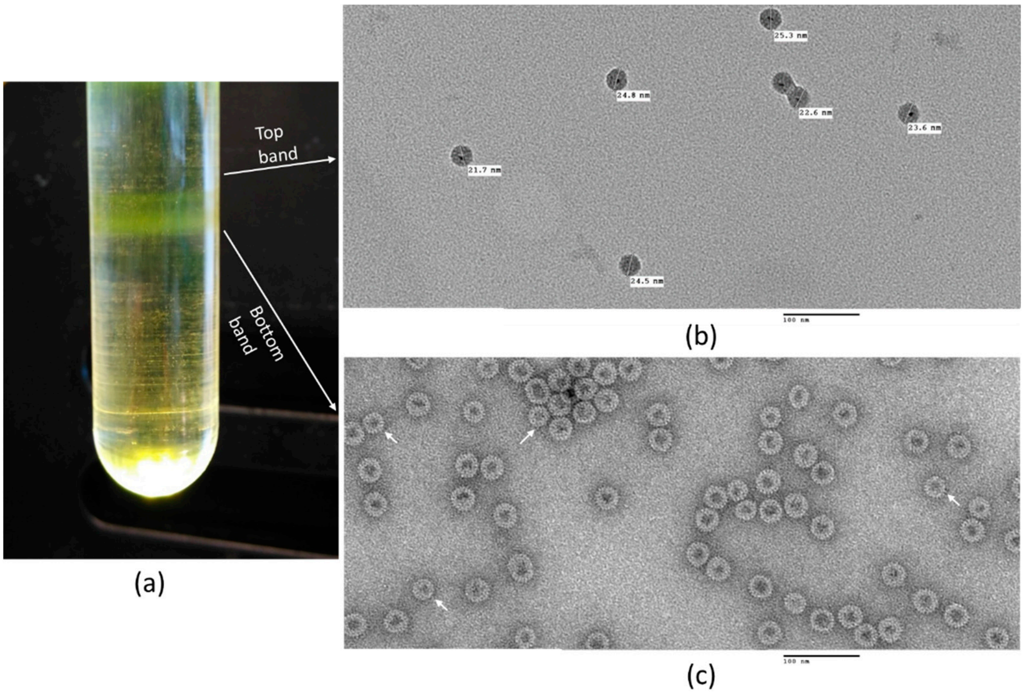

Figure S1. Nycodenz gradient purification and transmission electron microscopy (TEM) for the detection of HBcAg VLPs. (**a**) Visualization of two bands in Nycodenz gradient by downward illumination of the tube; (**b**) Negative stain TEM of the top band from Nycodenz gradient; (**c**) Negative stain TEM of bottom band from Nycodenz gradient. Scale bar = 100 nm. Arrows indicate smaller particles.

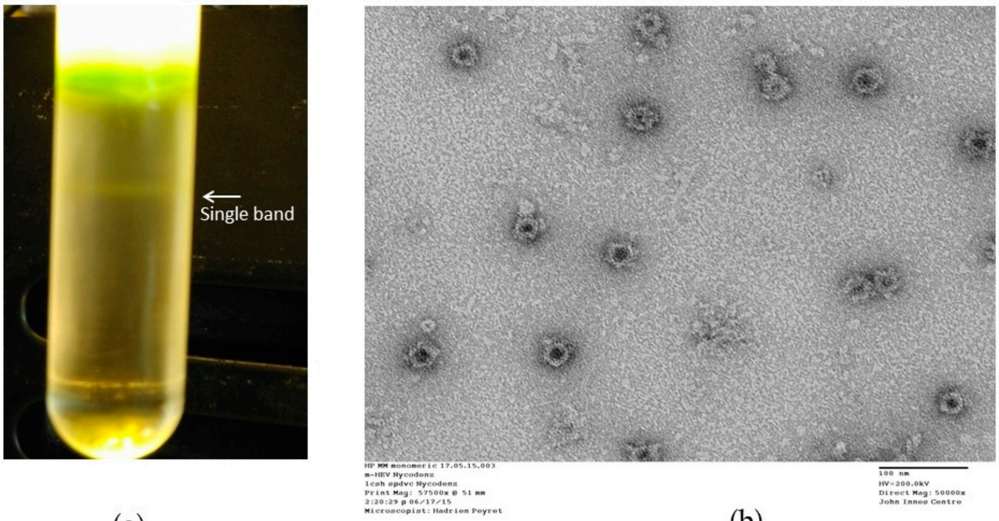

(a) (b)

Figure S2. Nycodenz gradient purification and transmission electron microscopy (TEM) detection of HBcHEV ORF2 551–607 VLPs. (**a**) Visible single band of VLPs observed from Nycodenz gradient; (**b**) TEM imaging of the collected single band. Particles were visualized by negative staining with uranyl acetate. Scale bar = 100 nm.
